# Evaluation of Trace Element and Metal Accumulation and Edibility Risk Associated with Consumption of *Labeo umbratus* from the Vaal Dam, South Africa

**DOI:** 10.3390/ijerph14070678

**Published:** 2017-06-23

**Authors:** Beric M. Gilbert, Ebrahim Hussain, Franz Jirsa, Annemariè Avenant-Oldewage

**Affiliations:** 1 Department of Zoology, University of Johannesburg, P.O. Box 524, Auckland Park, Johannesburg 2006, South Africa; bericg@uj.ac.za (B.M.G.); ebrahim.hussain@aucklandcouncil.govt.nz (E.H.); franz.jirsa@univie.ac.at (F.J.); 2 Institute of Inorganic Chemistry, University of Vienna, Waehringer Strasse 42, Vienna 1090, Austria

**Keywords:** biomonitoring, edibility, *Labeo umbratus*, trace element accumulation, Vaal River, Mercury and Arsenic

## Abstract

With the occurrence of recreational and small scale subsistence fishing activities at the Vaal Dam, South Africa, consumption of fish from this dam may result in health risks associated with trace elements and metals. The Vaal Dam is one of the largest dams in South Africa, located between the Gauteng Province and Orange Free State, and supplies water to approximately 11.6 million people. A total of 38 specimens of the benthic cyprinid fish *Labeo umbratus* were collected from the Vaal Dam during two surveys, in 2011 and 2016. Samples of muscle, liver, kidney, gill and spinal cord were analysed, along with sediment samples collected during the same surveys. Thirteen trace elements were analysed in the samples by Inductively Coupled Plasma–Optical Emission Spectrometry, Inductively Coupled Plasma–Mass Spectrometry, Atomic Absorption Spectroscopy and Total Reflection X-ray Fluorescence spectroscopy. This is the first survey on trace element and Hg accumulation in this fish species from the Vaal Dam and target hazard quotients (THQ) indicated that there is a risk for consumers of fish for As and Hg (THQ = 1.43 and 1.14 respectively). Although levels of trace elements in this impoundment have shown little change for a number of years and are lower than global background levels, studies detailing the accumulation of metals by fish inhabiting the Vaal Dam have indicated that trace elements in muscle tissue are above food safety guidelines. Trace element levels in *L. umbratus* are lower compared to other species inhabiting the Vaal Dam and further indicate that risks for consumers can be decreased if humans relying on fish from the Vaal Dam preferentially consume this species over others.

## 1. Introduction

Continual expansion of human populations has led to an almost unimaginable amount of waste being released daily into the environment. Aquatic ecosystems are especially at risk due to the fact that water is an indispensable resource required for anthropogenic processes and biological functions [1]. For this reason, monitoring of the chemical state and biological integrity of aquatic ecosystems is required to preserve this resource particularly in South Africa; a water-scarce country. The use of bioindicators in determining ecosystem health is superior to traditional measurement of physico-chemical variables alone [2,3,4,5,6,7], providing information on integrated influences and toxicity of pollutants to biota and ecosystems [8]. Although a number of organisms have been proposed as suitable bioindicators, much attention has been directed toward fish.

The suitability of fish as bioindicators was outlined by Kleynhans and Louw [9] as they are ubiquitous in most aquatic ecosystems, occupying upper trophic levels, are easily identifiable and have a long life span. In terms of toxicant accumulation, quantification of trace elements (e.g., As, Se, Zn) and metals (e.g., Cd, Cr, Pb) in a large number of fish species has been performed in both marine and aquatic habitats. Generally, metals and trace elements have been shown to accumulate mostly in particular organs such as the liver and gills, which has been related to the function of these organs. Along with the trend of analyzing trace element and metal bioaccumulation in fish, edibility studies have been conducted to determine potential health effects associated with consumption of fish exposed to toxicants in aquatic ecosystems [10,11,12,13].

The 1300 km-long Vaal River and its associated catchment area (38,500 km^2^) is considered South Africa’s hardest working river, supplying water to the economic hub of the country, which has an average population of over 10 million people [14,15]. This catchment area supplies water to the highly industrialized Pretoria-Witwatersrand-Vereeniging complex (PWV), as well as four of South Africa’s major provinces, namely, the Free State, Gauteng, North West and Mpumalanga [14,16]. The vast amount of industrial and agricultural activity within the Vaal River catchment area leads to large volumes of metal-containing waste (e.g., Mn, Zn and Sn), as well as urban and rural runoff being discharged into the system [17,18,19]. A number of studies have been conducted and assessed accumulation of metals by fish in the Vaal River and the Vaal Dam reservoir [10,20,21,22,23]. Possible sources of pollution in the Vaal Dam which could contribute to exposure to elevated trace element and metal levels have been linked to the presence of informal settlements and agricultural operations. Fewer studies have, however, investigated health risks associated with consumption of fish from the Vaal Dam [10,13]. Specifically with regard to species of *Labeo* from the Vaal Dam, Wepener et al. [24] and Lynch et al. [13] indicated that *Labeo capensis* (Orange River mudfish) was a suitable bioindicator for measurement of trace elements and metals in the Vaal River system. Furthermore, Lynch et al. [13] found that health risks were associated with the consumption of flesh of *L. capensis* from the Vaal Dam. As for *Labeo umbratus*, commonly known as moggel, until now no investigation has been conducted to assess the trace element content and edibility of this fish species. *Labeo umbratus* is a benthic species, feeding mostly on detritus, and is widely distributed throughout the Orange River and Vaal River systems [25].

The aim of this study was to analyse trace element content in the muscle, liver, kidney, gills and spinal cord of *L. umbratus*, from the Vaal Dam. Using the data collected for trace elements in muscle tissue, a preliminary health risk assessment was conducted in an attempt to determine if any risk existed with the consumption of *L. umbratus* from the Vaal Dam. Results of the analysis indicated that elements are differentially accumulated between organs of *L. umbratus* and health risks for Hg and As are associated with the consumption of muscle tissue.

## 2. Materials and Methods

The study was approved by the Ethics committee of the Faculty of Science at the University of Johannesburg and was conducted in accordance to all ethical requirements regarding animals involved in the study. Sampling was performed in accordance to appropriate permits for the collection of fish from the Gauteng Department of Agriculture and Rural Development (CPE3-000123).

**Figure 1:**
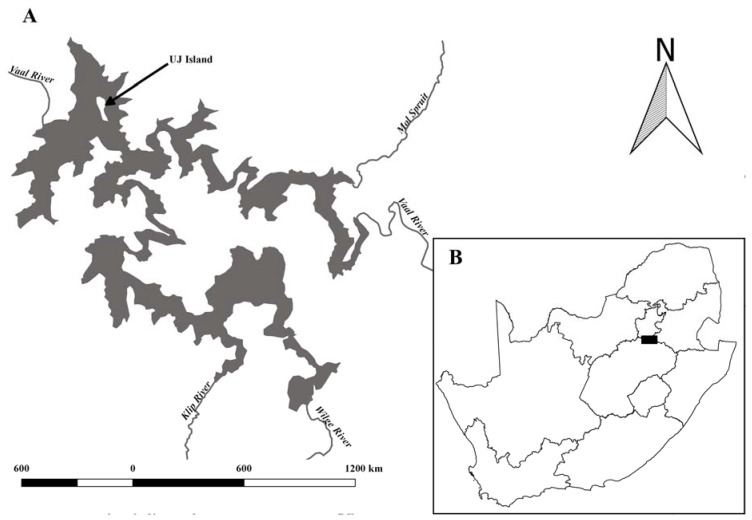
Location of the study site in the Vaal Dam (**A**). Insert (**B**) indicates location of the Vaal Dam in South Africa (black block).

Two surveys were conducted around the shores of the UJ Island (26°53′41″ S 28°08′44″ E) in the Vaal Dam, South Africa, which is situated approximately 60 km south of Johannesburg (Figure 1). *Labeo umbratus* collected using gill nets (mesh size: 70–110 mm) were removed and placed into aerated tanks at a field laboratory on the island. A total of 40 *L. umbratus* were collected; 28 February 2011 and 12 January 2016. All fish were weighed and measured before being euthanized by dislocation of the spinal cord, behind the head. During the 2011 survey, fish were then dissected using stainless steel forceps and scalpels. Gills (with gill arch), muscle, liver, kidney and spinal cord (with vertebrae) were removed, frozen at −20 °C and transported back to the laboratory. Three composite sediment samples were taken from the Vaal Dam using a grab sampler approximately 10 m from the shore in water approximately 2.5 m deep; these were frozen (−20 °C) until further analysis.

During the second survey, conducted in January 2016, muscle and liver samples were removed from adult *L*. *umbratus* post mortem using plastic forceps and a ceramic knife. The samples were then placed into sterile 50 mL centrifuge tubes, frozen at −20 °C and later transported back to the laboratory. Five samples each of surface sediment were collected from the east and west banks of UJ Island using 50 mL centrifuge tubes. The samples were taken 100 m apart, at a depth of approximately 70 cm.

### 2.1. Sample Preparation

#### 2.1.1. 2011 Samples

Tissue samples were defrosted and 1 g [wet weight (ww)] of each organ was dried to a consistent weight in an oven at 100 °C. The dry tissue samples were digested with 7 mL of 65% Suprapur^®^ nitric acid (Merck, Darmstadt, Germany) using an Ethos Touch Control advanced microwave station. Digested samples were decanted into 50 mL acid washed volumetric flasks, 100 µL of in standard solution (Merck, Darmstadt, Germany, 1000 mg/L in 2.3% *v/v* HNO_3_) was added and then brought to volume with Milli Q water. These diluted samples were then decanted into sterile 50 mL centrifuge tubes (Cellstar^®^ Tubes, Greiner Bio-One, Frickenhausen, Germany) and stored at 4 °C until further analyses.

Sediment samples were allowed to defrost and 1 g (ww) of sample was weighed out and dried similarly to the *L*. *umbratus* tissue samples. The dried sediment samples were leached with aquaregia (9 mL of 30% Suprapur^®^ hydrochloric acid (Merck, Darmstadt, Germany) and 3 mL of 65% Suprapur^®^ nitric acid (Merck, Darmstadt, Germany)) in an Ethos Touch Control advanced microwave station. Leachates were poured through filter paper (MN 615 number 1 filter papers) with a pore size of 4 μm (Marcherey–Nagel, Düren, Germany) to remove remaining silicate and decanted into 50 mL acid washed volumetric flasks; 100 µL In and Ga standard solutions (Inorganic Ventures, Christiansburg, VA, USA, 1000 μg/mL in 7% *v/v* HNO_3_) were added as internal standards and the samples were brought to volume with Milli Q water.

#### 2.1.2. 2016 Samples

Fish and sediment samples collected during the 2016 survey were freeze dried to weight consistency at −77 °C under negative pressure (−80 kPa) and were then transported to the laboratory in Vienna. Dried samples were leached/digested in an identical manner as described above for the 2011 samples, except only nitric acid (38%) was used for digestion of fish organ samples and made to a final volume of 20 mL with Milli Q water. In addition, reference samples were prepared by treating 0.2 g (dry weight) of fish protein DORM-3 and fish liver DOLT-5 obtained from the National Research Council Canada (NRCC, Ottawa, ON, Canada) in the same manner as described above for fish tissues.

### 2.2. Metal and Trace Element Analysis

The digested fish tissue samples collected during the 2011 survey were analysed by Rand Water using an Inductively Coupled Plasma–Mass Spectrometer (ICP-MS, X Series Thermo Elemental—Thermo Element Corporation, Thermo Fisher, Waltham, MA, USA) situated at the Rand Water analytical facility in Vereeniging. Sediment samples were analysed at the University of Johannesburg using an Inductively Coupled Plasma—Optical Emission Spectrometer (ICP-OES, SPECTRO ARCOS FH S12, SPECTRO Analytical Instruments GmbH, Kleve, Germany) for Cr, Mn, Fe and Cu and an Inductively Coupled Plasma—Mass Spectrometer (ICP-MS, SCIEX ELAN 6100, Perkin Elmer Inc., Waltham, MA, USA) for Ni, Zn, As, Se and Cd, which were below the detection limit of the ICP-OES.

Measurement of trace elements and metals in liver and muscle of *L. umbratus*, and sediment collected in 2016 was conducted in the following manner. Concentrations of As, Cu, Fe, Mn, Se, Sr, and Zn were determined by Total Reflection X-ray Fluorescence spectrometry (S2 PicoFox TXRF, Bruker Nano GmbH, Berlin, Germany). Cd, Cr and Ni levels were measured by graphite furnace atomic absorption spectrometry (PinAAcle 900Z GF-AAS, Perkin Elmer Inc., Waltham, MA, USA) and Hg determination was performed by Cold Vapor Atomic Absorption Spectrometry (FIMS 400 CV-AAS, Perkin Elmer Inc., Waltham, MA, USA). For all the measurements, samples were diluted with Millipore water where necessary. For the determination of the detection limits, analytical blanks were prepared without insertion of a sample. Recovery rates for the TXRF analyses are presented in Table S1, and respective recovery rates for GF-AAS and CV-AAS are given elsewhere [26].

### 2.3. Statistical Analysis

Analysis of all data was done using IBM SPSS Statistics version 20 (Statistical Package for the Social Sciences, SPSS Inc., Armonk, NY, USA). Homogeneity of data was assessed using the Shapiro-Wilk normality test and as data was found to be non-homogeneous, all element levels were log transformed by calculating the natural logarithm plus 1 [Ln (element concentration+1)]. Comparison of trace element levels between the different organs from *L. umbratus* collected during the two surveys was done using the Kruskal-Wallis test to determine the statistical significance of the distribution of elements between different *L. umbratus* organs and sediments analysed. Differences in trace element levels in muscle and liver from *L. umbratus* and sediments collected from the Vaal Dam were compared between surveys using the Mann-Whitney U test. The significance value was set at a 95% confidence range (*p* < 0.05). Calculation of the mean Se:Hg and mean Se:As molar ratio was done according to [27] where the concentration of the elements (in mg/kg) were divided by the atomic mass of Hg (200.59 g/mol), Se (78.96 g/mol) and As (74.922 g/mol) respectively.

### 2.4. Health Risk Assessment

Dry weight trace element and metal levels determined in muscle tissue of *L*. *umbratus* were converted to wet weight concentrations by multiplying by a factor of 0.2. This was done in accordance with the US Environmental Protection Agency [28] and Heath et al. [29] which have provided limits of trace elements and metals in fish tissues in wet weight levels. As there is no data available on the amount of fish consumed by people living around the Vaal Dam and in South Africa in general, the parameters used for calculation of the target hazard quotient (THQ) for each element were done according to the prescribed values suggested by the US EPA [28], which are indicated in Table 1. Further to analysis of current risk factors for *L. umbratus* tissue, the amount of fish necessary to be consumed to produce a THQ value of 1 was calculated for the given element levels.

## 3. Results

### 3.1. Sediment and Fish Tissue Trace Element Concentrations

During both surveys conducted, concentrations of Cr, Mn, Fe and Ni were higher in sediment compared to the organs of *L. umbratus* (Kruskall-Wallis; all *p* < 0.05). Comparison of mean element concentrations between sediment samples collected in 2011 and 2016 were insignificant for most elements (Cd, Cr, Fe, Mn, Ni and Zn; Mann-Whitney U; all *p* > 0.05) with only levels of As and Cu differing significantly (Mann-Whitney U; *p* < 0.05).

Variability in trace element concentrations in *L*. *umbratus* collected in 2011 indicated that most elements accumulated in the kidney and liver while lowest element levels were detected in muscle tissue. Of the trace elements determined in the organs of *L. umbratus* Cu > Zn > Fe were the highest and all accumulated in the liver. Overall, the sum of elements in the fish indicate that the highest element load was present in the liver, followed by the gills with arches > kidney > spinal cord with vertebrae > muscle. Comparison of the levels of trace elements in different organs analysed indicated that Fe accumulated higher than all other elements in the kidney, gills and spinal cord, whereas, in the liver the level of Cu was higher than other elements. In the muscle, Zn accumulated at the highest concentration. Differences in concentrations of all trace elements analysed were significant between muscle, liver, kidney, gills and spinal cord (Kruskall-Wallis; all *p* < 0.05). In most instances trace element levels in sediment were greater than in the fish tissue (Kruskall-Wallis; *p* > 0.05), except for As (Kruskall-Wallis *x*^2^ = 96.16, *p* = 3.41 × 10^−19^) and Cu (Kruskall-Wallis *x*^2^ = 108, *p* = 6.69 × 10^−22^), Se (Kruskall-Wallis *x*^2^ = 94.74, *p* = 6.78 × 10^−19^) and Zn (Kruskall-Wallis *x*^2^ = 54.2, *p* = 1.91 × 10^−10^) which were higher in kidney and liver respectively than in sediment.

For fish collected during the 2016 survey, only trace elements including metals in the muscle and liver of *L*. *umbratus* were analysed. Differences in trace element content were similar to the trends observed in fish from the 2011 survey and similarly indicate most elements accumulated in the liver, except for Hg which was higher in muscle. Differences between most element concentrations for liver and muscle from *L. umbratus* collected during 2016 were significant, except for Ni (Mann-Whitney *Z* = −1.59, *p* = 0.113). Differences between the trace element content of sediment and fish for 2016 indicated that, similar to the 2011 survey, most elements were significantly higher in sediment except for Cu (Kruskall-Wallis *x*^2^ = 13.55, *p* = 2.33 × 10^−4^) and Zn (Kruskall-Wallis *x*^2^ = 14.29, *p* = 1.57 × 10^−4^) which were higher in the liver compared to the sediment.

Comparison of the trace element content in liver and muscle between the 2011 and 2016 surveys indicated that in most instances, differences in trace element and metal concentrations were significant between surveys (Mann-Whitney U; all *p* < 0.05). However, the levels of some elements were not significantly different for different matrices between surveys and exceptions to this were observed for Cr, Fe, Mn, Ni and Zn in sediments; As, Cu, Fe, Ni, Se and Zn in liver; Fe and Zn in muscle (Table 2).

**Figure 2:**
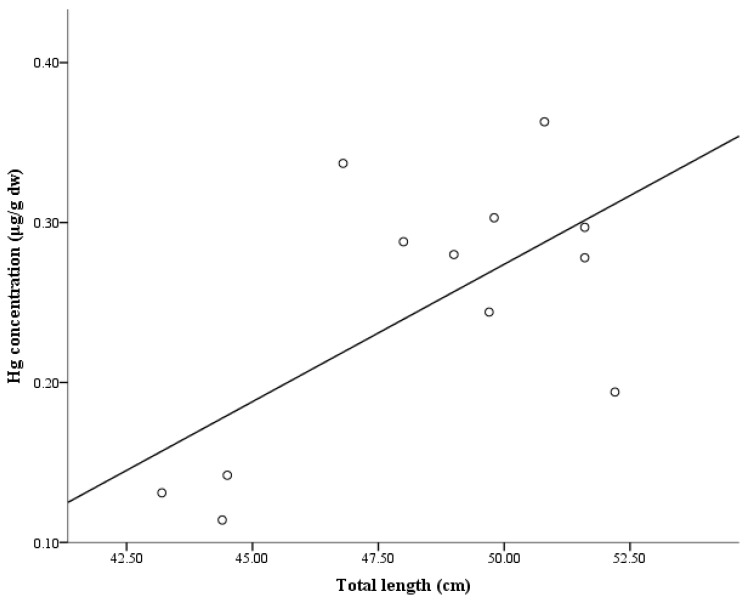
Regression curve indicating positive correlation between the mercury content in muscle tissue of *L. umbratus* and total length of the fish caught during 2016.

Determination of mean molar ratios for Se:Hg and Se:As in *L*. *umbratus* collected during 2016 indicate that none of the fish had Se:Hg ratios greater than that of the liver (mean ratio: 0.044). As the levels of both As and Se in muscle tissue were below detection, molar ratios could not be calculated for this organ. Similarly in liver tissue, low levels of As in this organ prevented calculation of As:Hg molar ratios. Accumulation of Hg in muscle tissue of *L. umbratus* was found to positively correlate with the total length of the fish sampled (Figure 2. *R*^2^ = 0.411). The positive correlation between mercury content and total length of *L. umbratus* was confirmed using a Pearson’s correlation and was significant (*r* = 0.641, *p* = 0.025).

### 3.2. Health Risk Assessment

The target hazard quotient for the elements measured in the muscle tissue of *L*. *umbratus* was determined, and indicated that health risks associated with the consumption of the flesh of *L*. *umbratus* were negligible, apart from As and Hg (Table 3) which indicated potential risks to humans eating the flesh of this fish species (Target Hazard Quotient (THQ) ≥ 1). Target hazards were calculated for adult individuals, including child-bearing women, according to the caveat indicated by the US EPA, which was an approximate weight of 70 kg and consumption of 54 g of fish daily for an entire year (365 days). Variation in the element concentrations between fish from different surveys were present and similarly THQ values differed. Comparison of THQ values calculated for all elements analysed in muscle tissue of *L. umbratus* from the two surveys indicated that generally, greater risk (values exceeding or equal to 1) was associated with fish caught during the 2011 survey compared to the 2016 survey. Only Cu and Hg were found to have higher THQ values in 2016 fish compared to *L. umbratus* sampled in 2011. As (1.43) and Hg (1.14) were found to pose potential health risks for people eating *L. umbratus* caught in the Vaal Dam (THQ ≥ 1). Furthermore, risk associated with As contaminated *L. umbratus* flesh was associated with 2011 caught fish, whereas Hg was only associated with fish caught in 2016.

At the current levels of trace elements and metals found in the flesh of *L. umbratus* sampled during both 2011 and 2016 surveys, a second consumption value (IRF_b_) was determined to ascertain the amount of fish muscle tissue which would have to be consumed in order to produce a minimum THQ value of 1 and is therefore, based on elements which did not pose any negative effect to human health in either of the two surveys. This new IRF_b_ indicated that on average, a mean of 0.837 kg/day of *L. umbratus* caught during 2011 would have to be consumed in order for all elements analysed to pose health risks. Whereas, for fish caught during 2016, a mean amount of 0.531 kg/day would have to be consumed to pose a risk to human health. Furthermore, there is little difference between the mean IRF_b_ for each survey, but comparatively larger amounts of fish caught during 2011 would have to be consumed compared to fish caught during 2016. The lower IRF_b_ value for 2016 correlates to the higher element levels determined in fish from this survey compared to the 2011 survey.

## 4. Discussion

### 4.1. Sediment and Fish Element Levels

Trace elements and metals in the Vaal Dam appear to have a low bioavailability, with sediments being enriched with higher levels of elements compared to a number of fish species inhabiting this impoundment. The results of the present study indicate trace element accumulation in *Labeo umbratus* and sediments collected from the Vaal Dam during two surveys in 2011 and 2016. Levels of trace elements and metals in the sediment samples from the Vaal Dam for both sampling periods are not significantly different to levels reported in previous studies and therefore indicate that there is little input of metals from sources of pollution in this impoundment [21,22,23,24,28]. However, this is not the case for the entire system, as indicated in previous studies which have shown that water quality and trace element levels along the length of the Vaal River are variable and can be related to human activities [23,24,28,30].

The concept of trace element bioaccumulation in fish is well known [31,32,33,34]. Trace element levels in the Vaal Dam have been assessed in a number of studies and findings in the present work corroborate trends previously observed. Furthermore, it demonstrates that trace elements are variably accumulated between the different organs of *L*. *umbratus*; the gills, liver and kidney being most active in this process and muscle and spinal cord demonstrating a poor accumulation potential for most of the trace elements analysed. This was similarly indicated for *Clarias gariepinus* by Crafford and Avenant-Oldewage [21,22], *Labeobarbus kimberleyensis* by Retief et al. [20,35] and Gilbert and Avenant-Oldewage [10], and in *Labeo capensis* by Lynch et al. [13] which similarly inhabit the Vaal Dam. This study therefore represents the latest in a series of studies to have analysed trace element levels in fish from the Vaal Dam and is the first report for *L. umbratus* in this system.

Although trace elements were present in most of the organs analysed, clear trends in accumulation were detected. From both 2011 and 2016 surveys it was evident that greater enrichment in the liver of *L*. *umbratus* by trace elements and metals occurred, and corroborated previous studies [29,33,34,35,36]. This trend can be related to the physiological function of the liver, as a principle detoxification site in fish [37,38,39,40]. Higher levels of Fe and Zn than other elements occurred in all organs of *L. umbratus* and likely relates to the essentiality of these elements as cofactors in fishes [41]. Along with these elements higher levels of Cu and Se were present in liver samples compared to other organs. In gills, Cr, Mn and Sr concentrations were higher than in other organs. In the kidney As and Ni were higher than other organs and Hg in muscle tissue. The trends observed in trace element and metal distribution in *L. umbratus* are similar to those reported for *L. umbratus* and other *Labeo* species from other river systems in South Africa [36,39] and the Vaal Dam [13,24].

Chromium levels in *L. umbratus* from the present study in the gill tissue corroborate with findings by Lynch et al. [13] who indicated higher Cr concentration in gill filaments of *L. capensis* (1.42 mg/kg). Whereas, Nussey et al. [39] in a study in the Olifants River indicated this element was higher in the liver (February 1994: Cr = 28.05 μg/g; February 1995: Cr = 66.21 μg/g) of *L. umbratus* and only occurred second highest in the gills. In February 1995, Coetzee et al. [36] found Cr was highest in the gills of *L. umbratus* inhabiting the Klein Olifants River (28 μg/g), but for fish collected in the Olifants River, this element was highest in the liver (58 μg/g), which agrees with the finding by Nussey et al. [39]. Higher Mn concentrations in the gills confirms findings by Coetzee et al. [36], Nussey et al. [39] and Lynch et al. [13]. Coetzee et al. [36] found that Mn levels were consistently higher in gill tissue of *L. umbratus* from the two sampling sites in their study (Mn: Klein Olifants River = 60 μg/g; Olifants River = 80 μg/g). Nussey et al. [39] similarly found that this was consistent between February 1994 (Mn = 105.67 μg/g) and February 1995 (Mn = 134.02 μg/g), and Lynch et al. [13] further indicated Mn to be associated most with gill filaments (Mn = 65.25 mg/kg) compared to the arches (Mn = 54.63 mg/kg) of *L. capensis*. In *Labeobarbus marequensis* higher Mn levels in gill arches were associated with binding of the element to Ca binding sites in the boney structures [42].

Higher concentrations of Cu, Fe, Se and Zn in the liver of *L. umbratus* supported findings by Crafford and Avenant-Oldewage [21,22] for *C. gariepinus*, and Retief et al. [20] and Gilbert and Avenant-Oldewage [10] for *L. kimberleyensis*, all of which were collected in the Vaal Dam. Visnjic-Jeftic et al. [43] also indicated higher levels of Cu, Fe and Zn in liver tissue of *Alosa immaculata*. Otachi et al. [44] also found higher Cu (470 mg/kg) and Fe (1930 mg/kg) levels in liver of *Oreochromis leucostictus* from Lake Naivasha in Kenya. For Zn, however, they found higher levels in muscle tissue (604 mg/kg) of *O. leucostictus* compared to other organs.

Higher levels of As and Ni in kidney tissue of *L. umbratus* corroborate previous findings, both for other fishes in the Vaal Dam [10] and from other aquatic systems [45,46]. Fargar et al. [45] found that As levels in the kidney (2.26 μg/g) of brown trout were greater than in other organs. Palaniappan and Karthikeyan [46] demonstrated a similar trend in Ni (116.82 μg/g) accumulation in the kidney compared to liver (Ni = 96.31 μg/g) of *Cirrhinus mrigala* through laboratory exposure. However, other studies have indicated a different trend, with As accumulating predominantly in the gills and muscle of fish [32,47]. Gilbert and Avenant-Oldewage et al. [10] attributed higher levels of trace elements in kidney tissue of *L. kimberleyensis* to the secondary function the kidney plays to the liver in detoxification of blood.

Comparison between gill and spinal cord tissue in the present study indicated that As and Co levels were relatively similar in gills and spinal cord of *L*. *umbratus* collected during 2011 survey. This finding may be related to the incorporation of these elements into the bony fraction of these organs (gill arch and vertebrae) during the calcification process [48,49]. These elements may, therefore, actively replace Ca in the bone matrix [48,49].

Muscle and spinal cord tissue overall demonstrated a poor accumulation capacity for most trace elements analysed, which accumulated at lower concentrations compared to other organs and can be related to the lower metabolic activity of these structures [32]. The accumulation of Hg at higher concentrations in muscle tissue has been well documented [47] and therefore higher Hg levels in muscle tissue of *L. umbratus* corroborates previous studies and can be related to the form of Hg in the environment [50]. Interest in this element stems from the health risks to humans associated with ingestion of fish contaminated with high levels of mercury, particularly methyl-mercury [51], as well as the bio-magnification of Hg through the trophic system [52]. This study is the first to report on Hg accumulation in fish from the Vaal Dam and therefore suggests possible health risks associated with the consumption of this fish species.

### 4.2. Health Risk Assessment

Edibility of a number of *Labeo* species from aquatic ecosystems in South Africa have been investigated and in most instances Cr, As, Sb and Pb have been found to pose health risks to people consuming the muscle tissue of these fishes as a means of subsistence [12,13,53]. In the present study most elements analysed did not pose any health risks associated with consumption of fish tissue by humans, except for As and Hg which had THQ values greater than one. Jooste et al. [12] reported that levels of Cr and Pb in muscle tissue of *L. rosae* from the Flag Boshielo Dam (THQ_Cr_ = 1.15; THQ_Pb_ = 4.18) and the Phalaborwa Barrage (THQ_Pb_ = 1.50) posed risks to human health. Lebepe et al. [53] analysed muscle samples of *L. rosae* from Flag Boshielo Dam and Loskop Dam, and determined that health risks were associated with contamination by Sb and Pb (Flag Boshielo Dam: THQ_Sb_ = 1.61; THQ_Pb_ = 1.84 and Loskop Dam: THQ_Sb_ = 21.9; THQ_Pb_ = 2.32). For *L. umbratus* inhabiting the Vaal Dam Cr did not pose risks to human health, whereas As and Hg did. Risk associated with consumption of fish flesh contaminated with As was similarly found by Lynch et al. [13] in *L. capensis* from the Vaal Dam, but the risk level determined by these authors far exceeded the risk determined in the present study even though tissue samples were prepared using a similar method. In the case of Hg, this study is the first to quantify health risks associated with this elements from a fish species in the Vaal Dam.

In a review of the environmental distribution of methyl-mercury and total mercury in selected water management areas (WMA) in South Africa, Walters et al. [54] indicated that the Upper Vaal WMA is a “hot spot” for Hg contamination by power stations and the main form which fish and other aquatic organisms are exposed to is methylated mercury. Compared to the prescribed safety limits for Hg in fish tissue according to the US EPA [55], levels in *L. umbratus* are lower (0.17 mg/kg ww) than 0.3 mg/kg [55]. Furthermore, compared to the classification suggested by Chvojka et al. [56] for Hg in fish muscle tissue, the levels determined in the muscle tissue of *L. umbratus* can be categorized as very low to low (0.05–0.25 μg/g).

Gilbert and Avenant-Oldewage [10] indicated that health risks were associated with the consumption of *L. kimberleyensis* for Cr (THQ = 1.5) and Se (THQ = 2.9), but found levels of As in *L. kimberleyensis* muscle tissue did not correspond with negative health risks. Interestingly, this comparison indicates that even though these fish species inhabit the same aquatic ecosystem, differences in the levels of trace elements and metals, and THQ values can possibly be related to the trophic position of these fishes. *Labeo umbratus* and *L. capensis* are both benthic fish species which feed on detritus and plant material, whereas *L. kimberleyensis* is an obligate piscivorous fish species [25]. An assessment of edibility of a number of fish species from aquatic ecosystems in Burkina Faso, Ouedraogo and Amyot [51] compared health risks associated with consumption of muscle from fishes of different trophic levels. They found that health risks associated with muscle tissue contaminated with Hg and As were generally lower in large piscivorous species compared to nonpiscivorous fish. This could be similar in the case of people eating fish from the Vaal Dam and consumption of fish should be restricted to piscivorous species compared to herbivorous species. However, further analysis of these trends is necessary to confirm this suggestion.

The calculation of consumption rates based on trace element and metal concentrations in *L. umbratus* from each survey showed that an average of 837 g and 531 g of fish collected during 2011 and 2016 respectively would have to be eaten to produce a THQ value of 1 for each element. Gilbert and Avenant-Oldewage [10] calculated that for most elements an average of 263 g/day of *L. kimberleyensis* muscle tissue would have to be consumed in order for most elements to pose a risk. For *L. umbratus* more muscle tissue (between 531 and 837 g/day) would have to be consumed to similarly produce a risk for elements which did not indicate any risk in the present study. This therefore, further serves as indication that humans relying on fish from the Vaal Dam should preferentially consume predatory species rather than herbivorous ones as the larger quantities of flesh needed to be consumed to produce equivalent health risks to predatory species (e.g., *L. kimberleyensis*) correlate with lower levels of elements. Furthermore, it is possible that the method of preparation may lower the risk factor associated with the consumption of contaminated fish flesh from aquatic ecosystems as indicated when comparing THQ values for elements in *L. umbratus* sampled in 2011 and 2016. Additional studies are, however, necessary and should analyse trace element and metal content in fish organs prepared using different methods, including traditional methods to ascertain if the method of preparation has any effect on risk factors.

The correlational relationship between Hg content and fish total length for *L. umbratus* from the Vaal Dam indicated a positive relationship between Hg levels in muscle and total length of the fish. Such an assessment is useful for the aquaculture industry as fish length is used as a factor to determine if fish are ready for sale to people for consumption purposes [57]. For *L. umbratus* it was shown that the level of Hg in fish muscle tissue was positively and significantly correlated with the total length of the fish. This implies that larger fish have higher Hg content than smaller fish. A number of studies have indicated such a positive correlation exists due to the trophic position and age of fish [57,58,59]. Therefore, as an advisory precaution to minimize exposure to Hg in *L. umbratus*, local people consuming this fish species should preferably consume smaller fish rather than larger, and older ones.

## 5. Conclusions

It was shown in the present study that trace element and metal levels in *L. umbratus* from the Vaal Dam are relatively lower compared to other fish species cohabiting this impoundment. For the first time it was shown that Hg accumulated to high levels in *L. umbratus* and due to the high health risk associated with this element, levels in other fish species in the Vaal Dam should also be determined. Similar analysis of trace elements and metals in sediment samples indicate that metals are below background levels and therefore do not indicate any signs of contamination. Along with this, the low bioavailability of trace elements in the Vaal Dam contributes to the relatively low risk factor associated with the consumption of trace elements and metals in the flesh of this fish species, with only As and Hg posing a risk to human health. In addition, in the case of variability of THQ values determined for *L. umbratus* collected during 2011 and 2016, lower THQs for fish collected in 2016 may provide some indication that the method of preparing fish flesh for consumption could potentially reduce the risk of harmful effects associated with eating contaminated muscle tissue. This was especially evident when comparing As in fish collected in 2011 where THQ values were greater than 1 but reduced to less than 1 in 2016. Therefore, it is suggested that further studies should be conducted to assess the effect of different methods of preparation on the trace element content of fish muscle tissue for consumption and therefore the edibility risk associated with contamination.

## Figures and Tables

**Table 1:** Parameters employed to calculate the target hazard quotient for determination of edibility of muscle tissue of *Labeo umbratus* collected from the Vaal Dam, South Africa according to the specified parameters by the US EPA [28] for adults, including pregnant women.

Average Weight (kg)	Average Time (Days)	Exposure Frequency (Days/Year)	Exposure Duration (Years)	Fish Consumption Weight (kg/Day)
BW_a_	AT	EF_r_	ED_r_	IRF_a_
70	365	350	30	0.054

**Table 2:** Mean (SD) trace element levels in water, sediment and muscle, liver, kidney, gills with arches and spinal cord with vertebrae of *Labeo umbratus* collected from the Vaal Dam, South Africa during 2011 and 2016 surveys. All trace element concentrations in sediment and fish organs have been expressed as dry weight levels. Means with common superscripts indicate no significant difference between surveys. ND = not determined in sample.

Element	Sediment 2011 *n* = 3 (mg/kg)	Sediment 2016 *n* = 10 (mg/kg)	Liver 2011 *n* = 28 (mg/kg)	Liver 2016 *n* = 12 (mg/kg)	Musc le 2011 *n* = 28 (mg/kg)	Musc le 2016 *n* = 12 (mg/kg)	Spinal Cord W. Vertebrae 2011 *n* = 28 (mg/kg)	Gills with Arc hes 2011 *n* = 28 (mg/kg)	Kidney 2011 *n* = 28 (mg/kg)
As	2.59 (1.28)	<4.75	3.25 (1.11)	6.33 (1.84)	0.927 (0.703)	<4.6	3.09 (2.57)	3.08 (0.979)	13.2 (10.7)
Cd	0.014 (0.006)	0.010 (0.010)	<0.11	0.460 (0.17)	<0.11	<0.006	<0.11	<0.11	<0.11
Cr	133 (182)	28.1 (14.4)	<0.01	0.130 (0.080)	1.67 (5.17)	0.049 (0.029)	2.08 (3.46)	2.79 (4.84)	0.412 (1.34)
Cu	11.9 (4.81)	6.13 (2.63)	844 (609)	1302 (823)	0.916 (0.592)	1.71 (0.703)	0.547 (0.281)	1.43 (0.836)	7.07 (5.06)
Hg	ND	0.01 (0.003)	ND	0.170 (0.050)	ND	0.247 (0.083)	ND	ND	ND
Fe	3586 (2070)	4832 (2928)	225 (232)	284 (103)	23.4 (72.3)	19.2 (30.3)	28.9 (44.4)	93.1 (80.4)	114 (138)
Mn	62.4 (32.1)	43.7 (28.1)	3.73 (3.10)	10.1 (7.60)	0.921 (0.378)	<4.6	11.4 (7.45)	14.2 (9.47)	1.33 (1.22)
Ni	12.1 (5.97)	7.28 (4.59)	0.395 (0.323)	0.14 (0.09)	0.360 (0.550)	<0.042	0.161 (0.146)	0.553 (0.950)	1.01 (1.11)
Se	0.346 (0.205)	<4.75	19.3 (18.2)	22.7 (6.60)	0.354 (0.285)	<4.60	0.208 (0.644)	0.563 (0.238)	0.725 (0.582)
Sr	ND	4.68 (1.51)	6.75 (2.57)	<4.60	17.7 (6.27)	12.5 (6.9)	256 (188)	293 (167)	6.32 (9.41)
Zn	21.0 (2.07)	13.2 (9.0)	257 (211)	222 (56)	32.1 (61.5)	16.9 (1.32)	30.4 (22.0)	71.9 (49.1)	45.6 (40.3)

**Table 3:** Target hazard quotient and recalculated consumption rates (IRF_b_) for each trace element measured in muscle tissue of *Labeo umbratus* from the Vaal Dam for 2011 and 2016 surveys. Concentrations of elements are based on mg/kg wet weights (ww) of tissue.

Survey	Variable	As	Cd	Cr	Cu	Fe	Hg	Mn	Ni	Se	Sr	Zn
	RfD_O_ (mg/kg/day)	0.0003	0.001	0.003	0.04	0.7	0.0001	0.14	0.02	0.005	0.6	0.3
2012	Element concentration (mg/kg; ww)	0.185	<0.11	0.334	0.183	4.68	-	0.184	0.0720	0.0708	3.54	6.42
	THQ	**1.43**	-	0.258	0.0106	0.0155	-	0.00305	0.00833	0.0328	0.0137	0.0495
	IRF_b_ (kg/day)	-	-	0.0210	0.509	0.349	-	1.77	0.648	0.165	0.395	0.109
2016	Element concentration (mg/kg; ww)	<4.60	<0.006	0.010	0.342	3.84	0.0494	<4.60	<0.042	<4.60	2.50	3.38
	THQ	-	-	0.00755	0.0198	0.0127	**1.14**	-	-	-	0.00964	0.0261
	IRF_b_ (kg/day)	-	-	0.716	0.273	0.425	-	-	-	-	0.560	0.207

Bold text indicate risk associated with a mean daily consumption of 54 g of *L. umbratus* muscle tissue; RfDo: Reference oral dosage; ww: wet weight; THQ: Target Hazard Quotient.

## Supplementary Materials

The following are available online at www.mdpi.com/1660-4601/14/7/678/s1, Table S1: Element concentrations in DOLT-5 certified by NRCC and as detected with TXRF: mean ± SD of nine measurements and mean accuracy all values in mg/kg dw.
