# Supplementary material for: Evaluation of Trace Element and Metal Accumulation and Edibility Risk Associated with Consumption of Labeo umbratus from the Vaal Dam, South Africa
Source: Int J Environ Res Public Health. 2017 Jun 23;14(7):678. doi: 10.3390/ijerph14070678 (PMC5551116; doi:10.3390/ijerph14070678)
Supplement: Supplementary file 1 [file ijerph-14-00678-s001.pdf]

Table S1: Element concentrations in DOLT-5 certified by NRCC and as detected with TXRF: mean ± SD of nine measurements and mean accuracy all values in mg/kg dw.

| Element | NRCC (mg/kg dw) ± SD | Measured Values (mg/kg dw) ± SD | Accuracy (%) |
|---|---|---|---|
| As | 34.6 ± 2.4 | 30.7 ± 0.6 | 88.7 |
| Cu | 35.0 ± 2.4 | 34.1 ± 0.8 | 97.4 |
| Fe | 1070 ± 80 | 1022 ± 18 | 95.5 |
| Mn | 8.91 ± 0.70 | 8.2 ± 0.5 | 92.0 |
| Se | 8.3 ±1.8 | 7.0 ±0.4 | 84.3 |
| Sr | 3.73 ± 0.26 | 4.0 ±0.3 | 107.2 |
| Zn | 105.3 ± 5.4 | 107.2 ± 5.5 | 101.8 |
